# Metabolomic Analysis of Small Extracellular Vesicles Derived from Pancreatic Cancer Cells Cultured under Normoxia and Hypoxia

**DOI:** 10.3390/metabo11040215

**Published:** 2021-04-01

**Authors:** Ryosuke Hayasaka, Sho Tabata, Masako Hasebe, Satsuki Ikeda, Sumiko Ohnuma, Masaru Mori, Tomoyoshi Soga, Masaru Tomita, Akiyoshi Hirayama

**Affiliations:** 1 Institute for Advanced Biosciences, Keio University, Tsuruoka, Yamagata 997-0052, Japan; ryosukeh@sfc.keio.ac.jp (R.H.); tabatasho@protein.osaka-u.ac.jp (S.T.); mhasebe@ttck.keio.ac.jp (M.H.); satsuki@ttck.keio.ac.jp (S.I.); o-su-mi@ttck.keio.ac.jp (S.O.); m-masaru@ttck.keio.ac.jp (M.M.); soga@sfc.keio.ac.jp (T.S.); mt@sfc.keio.ac.jp (M.T.); 2 Systems Biology Program, Graduate School of Media and Governance, Keio University, Fujisawa, Kanagawa 252-0882, Japan; 3 Institute for Protein Research, Osaka University, Suita, Osaka 565-0871, Japan; 4 Institute of Nano-Life-Systems, Institutes of Innovation for Future Society, Nagoya University, Nagoya, Aichi 464-8603, Japan

**Keywords:** small extracellular vesicles, metabolome analysis, pancreatic cancer, hypoxia, capillary ion chromatography-mass spectrometry, liquid chromatography-mass spectrometry, supercritical fluid chromatography-tandem mass spectrometry

## Abstract

Extracellular vesicles (EVs) released from cancer cells contribute to various malignant phenotypes of cancer, including metastasis, cachexia, and angiogenesis. Although DNA, mRNAs, miRNAs, and proteins contained in EVs have been extensively studied, the function of metabolites in EVs remains unclear. In this study, we performed a comprehensive metabolomic analysis of pancreatic cancer cells, PANC-1, cultured under different oxygen concentrations, and small EVs (sEVs) released from them, considering the fact that hypoxia contributes to the malignant behavior of cells in pancreatic cancer, which is a poorly diagnosed cancer. sEVs were collected by ultracentrifugation, and hydrophilic metabolites were analyzed using capillary ion chromatography-mass spectrometry and liquid chromatography-mass spectrometry, and lipids were analyzed by supercritical fluid chromatography-tandem mass spectrometry. A total of 140 hydrophilic metabolites and 494 lipids were detected in sEVs, and their profiles were different from those in cells. In addition, the metabolomic profile of sEVs was observed to change under hypoxic stress, and an increase in metabolites involved in angiogenesis was also detected. We reveal the hallmark of the metabolites contained in sEVs and the effect of tumor hypoxia on their profiles, which may help in understanding EV-mediated cancer malignancy.

## 1. Introduction

Extracellular vesicles (EVs) are lipid bilayer structures with a diameter of less than 1 μm; they include exosomes (50–150 nm) and microvesicles (100–1000 nm) [1]. EVs are contained in body fluids, such as blood, urine, and saliva, and are also present in the supernatant of cultured cells [1]. In addition, they play important roles in promoting cancer progression, such as in distant metastasis, proliferation, angiogenesis, and resistance to treatment [2,3,4,5]. EVs contain DNA, mRNAs, miRNAs, proteins, and lipids, and their amounts vary depending on the type of cell and physiological conditions. Among proteins, tetraspanins, such as CD9, CD63, and CD81, as well as syntenin-1 and Alix, are found to accumulate in EVs as compared to cells [6]. Moreover, in various cancer cell lines, lipids including cholesterol, sphingomyelin, glycosphingolipid, and phosphatidylserine are enriched in EVs, whereas phosphatidylcholine and phosphatidylinositol are less abundant [7,8,9,10]. Zhao et al. reported that EVs derived from cancer associated fibroblasts contain 34 hydrophilic metabolites, including organic acids and amino acids, some of which are metabolized by the recipient’s cells [11]. Therefore, some of the hydrophilic metabolites contained in EVs are possibly involved in the malignant transformation of cancer [11], but their details remain unclear.

Metabolomic analysis, which comprehensively analyzes metabolites in a given sample, is currently used in various fields, such as cancer metabolism and biomarker research [12,13,14]. As individual metabolites have a wide variety of physical and chemical properties, it is impossible to measure all the metabolites using a single method. Therefore, it is necessary to select an appropriate method for metabolite analysis. Gas chromatography-mass spectrometry, liquid chromatography-mass spectrometry (LC-MS), and capillary electrophoresis-mass spectrometry have been frequently employed as mass spectrometric methods for metabolomic analysis. Furthermore, in recent years, the development of novel analytical methods has made it possible to measure metabolites using different separation strategies. For example, ion chromatography-mass spectrometry (IC-MS) has been developed to analyze highly hydrophilic compounds [15,16,17,18]. We have also developed a capillary IC-MS method for metabolomic analysis and have reported its high selectivity and sensitivity for anionic metabolites, such as nucleotides, sugar phosphates, and organic acids [19]. In addition, supercritical fluid chromatography coupled with triple quadrupole mass spectrometry (SFC-QqQMS) has attracted attention as a novel lipid analysis method that can chromatographically separate individual lipid classes and quantify each lipid species without being significantly affected by ion suppression [20].

Pancreatic cancer is the fourth leading cause of cancer-related deaths in the USA [21] and Japan [22]. The three-year survival rate of pancreatic cancer in Japan is 15.1%, and it is known to be associated with the worst prognosis among all cancers [22]. The factors behind the poor prognosis in patients with pancreatic cancer include difficulty in detection at early stages, local infiltration, and rapid metastasis. Furthermore, hypoxia is also considered a cause of the poor prognosis of pancreatic cancer [23]. In various cancers, hypoxia provides a specific microenvironment. Hypoxic stress controls the expression of more than 800 genes involved in energy metabolism, cancer growth, angiogenesis, and apoptosis [24]. Metabolism-related genes are also among the genes whose levels are altered under hypoxic stress, which significantly changes the metabolism in cancer cells, aiding their survival [25].

Studies on EVs released from cells under hypoxic stress have been reported. In many cancer types, miR-210 is upregulated in both cells and EVs under hypoxic stress and is involved in angiogenesis [26]. EVs released under hypoxic stress are also involved in the inhibition of NK cell function, induction of macrophages to the M2 type, and regulation of T cell proliferation and differentiation in a specific cancer type [27,28,29,30]. However, the effect of hypoxic stress on the metabolomic profile of EVs remains unclear.

In this study, we comprehensively analyzed the metabolomic profile of cells and EVs derived from the human pancreatic cancer cell line, PANC-1, using three different methods for the analysis of metabolome: capillary IC-MS, LC-MS, and SFC-QqQMS. The target EVs were small EVs (sEVs) with a diameter less than 200 µm, which is closer to that of exosomes. We also investigated changes in the amount of metabolites in sEVs when cells were subjected to hypoxic stress. We found that the metabolomic profiles of cells and sEVs are different, and the levels of metabolites present in sEVs fluctuate under hypoxic stress.

## 2. Results

### 2.1. Isolation of sEVs Released from PANC-1 Cells Cultured under Normoxia and Hypoxia

**Figure 1:**
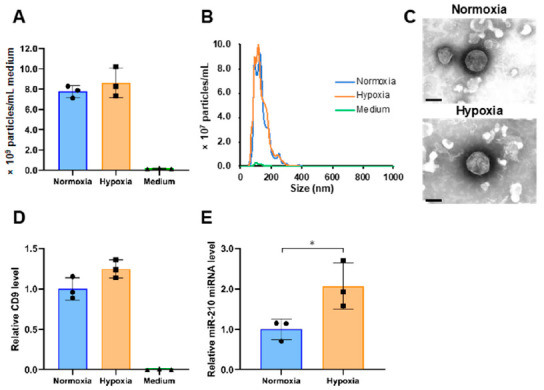
Characterization of small extracellular vesicles (sEVs) derived from PANC-1 cells cultured under different oxygen concentrations. (**A**) The number of particles in normoxia, hypoxia, and medium samples. (**B**) Particle size distribution in normoxia, hypoxia, and medium samples. (**C**) Transmission electron microscopic images of sEVs under normoxia and hypoxia. Scale bar, 100 nm. (**D**) The relative expression levels of CD9 in normoxia, hypoxia, and medium samples. (**E**) The expression level of miR-210 in normoxia and hypoxia samples. The expression data were normalized to the number of particles. Symbols indicate the result of each sample, and the bar graphs indicate the mean ± SD (*n* = 3). The Student’s *t*-test was used to determine the statistical significance. * *p* < 0.05.

We isolated the sEVs released from PANC-1 cells cultured under conditions of 20% (normoxia) or 1% (hypoxia) O_2_ by ultracentrifugation (234,700 × *g* for 70 min at 4 °C) of 260 mL of conditioned medium. As seen in Figure 1A, almost no particles were detected in the nanoparticle tracking analysis of the uncultured medium, but a considerable number of particles were observed under normoxia and hypoxia (normoxia, 7.8 × 10^9^/mL; hypoxia, 8.6 × 10^9^/mL; *p* = 0.41). The average particle sizes of sEVs obtained under normoxia and hypoxia were 135.6 and 134.1 nm, respectively (Figure 1B). Transmission electron microscopy revealed that the particles were round and had a lipid bilayer structure (Figure 1C). The levels of CD9, which is a marker protein for sEVs, were measured using ELISA. The level of CD9 under hypoxia was 1.2-times higher than under normoxia (Figure 1D). In addition, immunoblotting confirmed the presence of other sEV markers, including CD63, CD81 and syntenin-1, and the absence of endoplasmic reticulum (non-sEVs) marker, calnexin (Supplementary Figure S1). Furthermore, to verify the response to hypoxic stress in cells and sEVs, we examined the expression of genes and microRNAs that have been reported to be upregulated under hypoxia in cells or sEVs [24,26]. The induction of hypoxia-mediated genes in cells and of miR-210 in sEVs was confirmed (Supplementary Figure S2 and Figure 1E).

### 2.2. Analysis of Hydrophilic Metabolites

#### 2.2.1. Relationship of Hydrophilic Metabolites in Cells and sEVs

**Figure 2:**
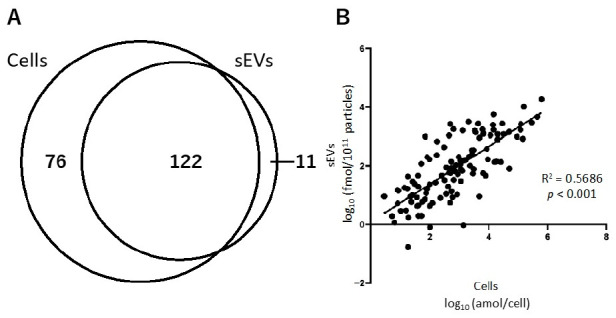
The relationship of hydrophilic metabolites present in cells and small extracellular vesicles (sEVs) obtained under normoxia. (**A**) Venn-diagram representing the number of hydrophilic metabolites detected in cell and sEV samples. (**B**) Correlation of the hydrophilic metabolites present in cells and sEVs.

We analyzed the hydrophilic metabolome of cells and sEVs using capillary IC-MS and LC-MS. For sEVs, to remove the influence of metabolites contained in the medium, uncultured medium was similarly pretreated and analyzed, and the amount of metabolites identified in it was subtracted from those identified in sEVs. A total of 198 and 133 hydrophilic metabolites were detected in more than 50% of cell and sEV samples under normoxia, respectively (Figure 2A). Of these, 122 metabolites were common in both the samples. Seventy-six metabolites, including amino acids, such as tryptophan, beta-alanine, and GABA, as well as lactic acid and deoxynucleotides were detected only in cells. In contrast, 11 metabolites, including ribonucleosides, such as cytidine, uridine, and guanosine, were detected only in sEVs (Supplementary Table S1). A scatter plot of 122 hydrophilic metabolites that were common in cells and sEVs showed a significant correlation between the two types of samples (R^2^ = 0.5686, *p* < 0.001; Figure 2B).

Next, we compared the top 20 most abundant metabolites in cells and sEVs (Table 1). Among these top 20 metabolites, seven, viz., phosphorylcholine, glycerophosphorylcholine, glutamic acid, ethanolamine phosphate, UDP-N-acetylglucosamine, glutamine, and glycine, were common in both the samples. Of the 11 metabolites detected only in sEVs, four compounds, namely inosine; *N*,*N*-dimethylglycine; cytidine and uridine, were included among the top 20 metabolites.

#### 2.2.2. Effect of Hypoxic Stress on the Level of Hydrophilic Metabolites in sEVs

**Figure 3:**
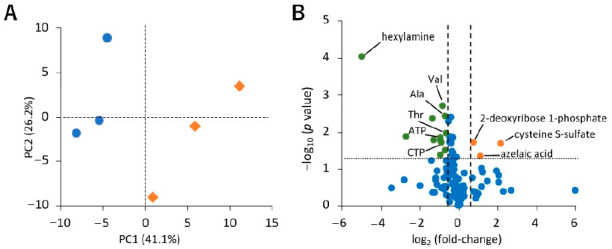
Effect of hypoxic stress on hydrophilic metabolite profiles in small extracellular vesicles (sEVs). (**A**) Principal component analysis (PCA) score plots for normoxia (blue circle) and hypoxia (orange diamond) samples based on hydrophilic metabolome data. The contribution ratios were 41.1% and 26.2% for PC1 and PC2, respectively. (**B**) Volcano plot showing the differential accumulation of metabolites under normoxia and hypoxia. The Student’s *t*-test was used to determine the *p* value. The plots marked in orange and green are for metabolites that were increased and decreased under hypoxia, respectively. Metabolites that were detected in over 50% samples were included for the analysis. Data were normalized by the number of particles, as quantified by NanoSight. As hexylamine was detected only under normoxia, its log_2_ (fold-change) value is displayed as −5, *n* = 3.

To investigate the differences in the levels of hydrophilic metabolites in sEVs under normoxia and hypoxia, we performed a principal component analysis (PCA) and volcano plot analysis. The normoxia and hypoxia samples were separated by principal component 1 (Figure 3A). The volcano plot shows that the amounts of 2-deoxyribose 1-phosphate, cysteine S-sulfate, and azelaic acid were increased (*p* < 0.05, fold-change > 1.5), and a decrease was observed for 12 metabolites, namely valine, alanine, tartaric acid, threonine, o-acetylcarnitine, ATP, Gly Leu, CTP, 2-oxoglutaric acid, cytidine, fumaric acid, and hexylamine (Figure 3B).

### 2.3. Lipid Analysis

**Figure 4:**
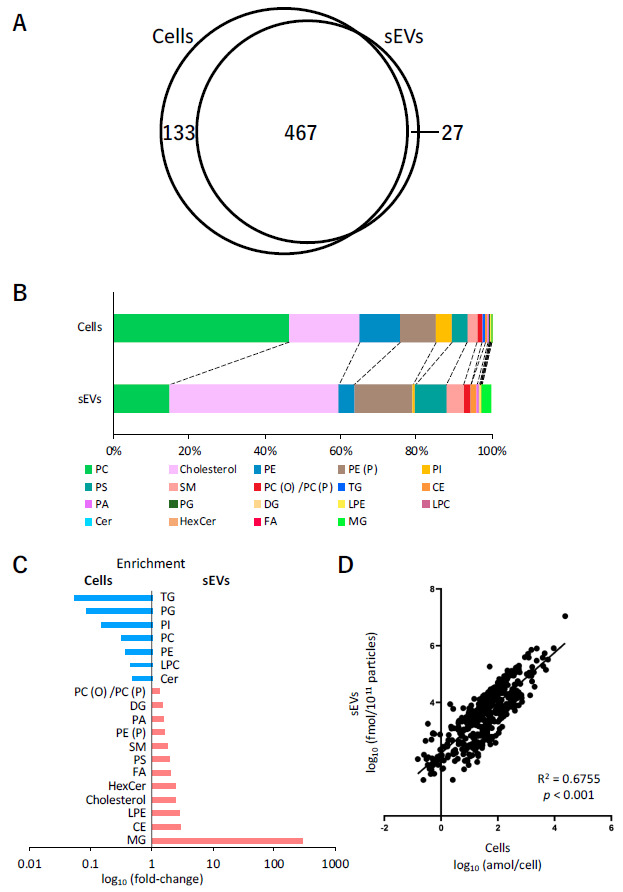
Lipidomic profiles of cells and small extracellular vesicles (sEVs) under normoxia. (**A**) Venn-diagram representing the number of lipids detected in cell and sEV samples. (**B**) Pie plots showing the mol-based percentile of each lipid class in cells and sEVs. (**C**) Enrichment of lipid classes in cells or sEVs. The data are shown as fold-changes of the ratio of lipid contents in sEVs per those of cells. (**D**) Correlation of the lipids in cells and sEVs.

Analysis of lipids in cells and sEVs was performed using SFC-QqQMS. Similarly to the measurement of hydrophilic metabolites, the amount of lipids in sEVs was calculated by subtracting the result obtained for the blank sample collected from the uncultured medium. A total of 600 and 494 lipids belonging to 19 lipid classes were identified in cells and sEVs, respectively, and 467 of these were common in both the samples (Figure 4A). Table 2 shows the number of lipids in each lipid class in the cell and sEV samples. The lipids that were only detected in cells were phosphatidylcholine (PC), phosphatidylethanolamine (PE), phosphatidylinositol (PI), and lysophosphatidylcholine (LPC), and of the 27 lipids detected only in sEVs, 19 were monoacylglycerol (MG) or diacylglycerol (DG).

Next, the relationship between the content of each of the lipid classes in cells and sEVs was examined. In cells, the content of PC (46.6%), cholesterol (18.5%), PE (10.9%), and alkenyl-acyl phosphatidylethanolamine (PE (P)) (9.4%) was relatively higher, whereas that of cholesterol (45.0%), PE (P) (15.4%), PC (14.7%), and phosphatidylserine (PS) (8.3%) was higher in sEVs (Figure 4B). A comparison of the ratio of each lipid class to the total lipid content showed that the levels of MG (296 times), cholesterol ester (CE) (2.9 times), lysophosphatidylethanolamine (LPE) (2.9 times), cholesterol (2.4 times), hexosylceramide (HexCer) (2.4 times), free fatty acid (FA) (2.1 times), and PS (2.0 times) increased more than two times in sEVs compared with their respective levels in cells. In contrast, the levels of triacylglycerol (TG) (0.05 times), phosphatidylglycerol (PG) (0.09 times), PI (0.2 times), PC (0.31 times), PE (0.37 times), LPC (0.43 times), and ceramide (Cer) (0.47 times) in sEVs decreased to less than half of their levels in the cells (Figure 4C). Furthermore, the relationship between individual lipids in cells and sEVs was also investigated. Figure 4D shows the differences in the levels of 467 lipids that were common in cells and sEVs. Overall, these profiles were adequately correlated (R^2^ = 0.6755, *p* < 0.001).

Finally, the difference in the amount of each lipid class in cells and sEVs between normoxia and hypoxia was investigated (Table 3). In cells, an increase (*p* < 0.05) was observed under hypoxic stress in 15 of the 19 lipid classes. However, no obvious effect of hypoxic stress on lipid classes was observed in sEVs.

## 3. Discussion

In this study, we performed a metabolomic analysis of PANC-1 cells cultured under normoxia and hypoxia and sEVs obtained from the culture supernatant of these cells by ultracentrifugation. Hypoxic stress has been reported to increase the release of sEVs from cells [29,31]. However, no apparent difference in the number of particles, particle size distribution, or the content of marker protein was observed in the presence and absence of hypoxic stress in this study. In a previous study using another pancreatic cancer cell line, AsPc-1, no difference was observed relative to the number of particles under hypoxic stress (1% O_2_) and 20% O_2_ conditions, but a significant increase was observed at 0.1% O_2_ [32]. Furthermore, in an experiment in which chronic myelogenous leukemia cells were subjected to hypoxic stress (1% O_2_) for 24 h, no increase was observed in the number of sEVs [4]. On the contrary, miR-210, a hypoxia marker gene, whose expression increases in various cancers [4,26,33], was also found to increase in this study. Therefore, we conclude that the sEVs analyzed in this study can be collected under sufficient hypoxic stress.

We could quantify 140 hydrophilic metabolites from sEVs by hydrophilic metabolome analysis using capillary IC-MS and LC-MS, and 494 lipids by lipidome analysis using SFC-QqQMS. There have been several reports on hydrophilic metabolites since it was first shown in 2015 that sEVs derived from bone marrow mesenchymal stem/stromal cells contain lactic acid and glutamic acid [11,34,35,36]. In the present study, we have succeeded in quantifying 140 hydrophilic metabolites, including amino acids and their derivatives, organic acids, sugar phosphates, and nucleotides, and this number is the largest compared to previous reports.

Of the 11 hydrophilic metabolites detected only in sEVs, inosine, guanosine, hypoxanthine, and xanthine are involved in purine metabolism, and cytidine and uridine are involved in pyrimidine metabolism. Furthermore, inosine, cytidine, and uridine were among the top 20 metabolites with the highest amounts in sEVs. The existence of intermediate metabolites of purine and pyrimidine metabolism in sEVs has already been reported. For example, sEVs derived from a breast cancer cell line were reported to have high concentrations of uridine and guanosine [37]. In addition, it has been reported that sEVs derived from head and neck squamous cell carcinoma cell lines and plasma from patients also contain intermediates for purine metabolism, which exhibit an immunosuppressive effect [38,39]. Taking these results into account, even in the sEVs derived from PANC-1, intermediate metabolites of purine and pyrimidine metabolism are actively incorporated into sEVs and might create a favorable environment for cancer metastasis and growth through immunosuppression.

We found a significant correlation for 122 hydrophilic metabolites that were commonly found between cells and sEVs. However, when comparing the top 20 hydrophilic metabolites with high amounts in cells and sEVs, only seven metabolites were common. This suggests that the concentration of hydrophilic metabolites in sEVs is not largely affected by their concentration in the cells from which they originate. There are several possible causes for this—for example, a specific metabolite is selectively taken up by the sEVs when it is produced into the cell, or it is metabolized or decomposed internally after the generation of sEVs. However, the detailed mechanism of uptake of metabolites by sEVs is unknown and needs further investigation in the future.

When PCA was performed using the quantitative values of hydrophilic metabolites in sEVs recovered under normoxia and hypoxia, it was found that both the samples were separated for principal component 1. In addition, volcano plots revealed changes in 15 metabolites (*p <* 0.05, fold-change < 0.67 or >1.5). Among these, 2-deoxyribose 1-phosphate, cysteine S-sulfate, and azelaic acid were increased in sEVs under hypoxic stress. In this study, 2-deoxyribose 1-phosphate was not detected in cells, but was only detected in sEVs. Thymidine phosphorylase (TP), uridine phosphorylase, and purine nucleoside phosphorylase are major enzymes involved in the production of 2-deoxyribose 1-phosphate [40]. TP promotes tumor cell proliferation in hypoxia and resists hypoxic stress-induced apoptosis [41]. In addition, 2-deoxyribose 1-phosphate promotes angiogenesis by attracting vascular epithelial cells in an NF-κB-dependent manner [42]. It has already been reported that the expression of many molecules, such as urothelial cancer associated 1 (UCA1), carbonic anhydrase 9 (CA9), tissue factor (TF), miR-135b, miR-23a, and miR-494, increases with hypoxia, and they are involved in angiogenesis [31,43]. Therefore, in addition to these molecules, 2-deoxyribose 1-phosphate may also be involved in the progression of cancer through the promotion of angiogenesis.

Cysteine S-sulfate is a precursor of the antioxidant cysteine. The concentrations of cysteine and cysteine S-sulfate decrease in the serum of glioma patients [44]. Azelaic acid is the final product of linoleic acid decomposed into peroxides [45] and is detected in the blood, feces, and saliva of patients with gastric cancer [46,47]. However, the relationship between these metabolites and pancreatic cancer and hypoxic stress is unclear. In contrast, hypoxic stress resulted in a decrease in valine, alanine, tartaric acid, threonine, o-acetylcarnitine, ATP, Gly Leu, CTP, 2-oxoglutaric acid, cytidine, fumaric acid, and hexylamine. Cell metabolism shifts from aerobic phosphorylation to glycolysis under hypoxic stress—a phenomenon that has long been known as the Warburg effect [48]. Moreover, in some cancers, hypoxic stress significantly changes amino acid metabolism and expression of some amino acid transporters [49,50,51]. These intracellular metabolic changes induced by hypoxic stress might have affected the metabolite content of sEVs.

Since the study conducted by Vidal et al. [52], a great deal of research has been performed on lipid analysis in sEVs [9,53,54,55]. The commonly observed phenomena in these studies were an increase in cholesterol, sphingomyelin (SM), and PS, and a decrease in PC and PI in sEVs as compared to cells. As similar trends were observed in our study, we consider that lipid analysis of sEVs performed by us was successful.

A comparison of the content of each lipid class in cells and sEVs revealed a decrease in TG and a substantial increase in DG and MG in sEVs. Additionally, the FA content increased by 2.1-times in sEVs, and several DGs and MGs, which were not detected in cells, were detected in sEVs. It may be possible that some kind of decomposition occurred in sEVs. In fact, it has been reported that sEVs derived from pancreatic cancer carry a bioactive peptide called adrenomedullin, which is involved in the breakdown of lipids in adipose tissue, and may contribute to the decomposition of lipids in sEVs [56]. Moreover, it has been also reported that MG is enriched in exomere, which is an extracellular particle less than 35 nm in diameter [57]. Although the proportion of the exomere fraction in the sEVs recovered in this experiment is unknown, it might contribute to the fluctuations in specific lipid classes.

As only a single pancreatic cancer cell line was used in this study, it was not possible to determine whether the metabolomic profile in sEVs observed here is common to pancreatic cancer. In addition, because the recovery rate of sEVs by ultracentrifugation was low, a large amount of culture supernatant (260 mL) was required. Owing to the limitations of the equipment used for the culture and processing, we could analyze only three biological replicates. In the future, if a method that can recover sEVs more efficiently is developed, it may be possible to perform a more detailed analysis by increasing the number of replicates.

In conclusion, we detected 140 hydrophilic metabolites and 494 lipids in sEVs derived from the pancreatic cancer cell line PANC-1, using a comprehensive metabolomic analysis method involving capillary IC-MS, LC-MS, and SFC-QqQMS. The profiles of hydrophilic metabolites and lipids in sEVs were found to be different from those in cells. The intermediates of purine and pyrimidine metabolism were found to be specifically accumulated in sEVs. Furthermore, it is suggested that the metabolomic profile of sEVs may be altered by hypoxic stress. These metabolites, which fluctuate in sEVs due to external stress, may have some involvement in cancer growth and metastasis.

## 4. Materials and Methods

### 4.1. Cell Culture

Human pancreatic cancer cells, PANC-1, were obtained from the American Type Culture Collection (ATCC, Manassas, VA, USA). PANC-1 cells were confirmed to be mycoplasma-free using a Mycoalert detection kit (Lonza, Basel, Switzerland). PANC-1 cells were cultured in RPMI 1640 (FUJIFILM Wako Pure Chemical Corporation, Osaka, Japan), supplemented with 10% (*v/v*) fetal bovine serum (FBS, Biowest, Nuaillé, France) and antibiotics (100 U/mL penicillin, 100 mg/mL streptomycin, and 0.25 mg/mL amphotericin B, Nacalai Tesque, Kyoto, Japan), at 37 °C in a humidified atmosphere with 5% CO_2_ and 20% O_2_.

### 4.2. Isolation of sEVs

For isolation of sEVs, 6.0 × 10^6^ PANC-1 cells were seeded in thirteen 150 mm dishes and precultured with RPMI 1640 containing FBS and antibiotics for 24 h. They were washed twice with Dulbecco’s phosphate-buffered saline (D-PBS, Nacalai Tesque). As FBS also includes large amounts of sEVs, it is necessary to change to advanced RPMI 1640 medium (Thermo Fisher Scientific, Waltham, MA, USA) containing 2 mmol/L glutamine (Thermo Fisher Scientific) and antibiotics. Thereafter, the cells were cultured for 48 h under hypoxia (1% O_2_) or normal oxygen conditions (*n* = 3). Fresh (non-cultured) advanced RPMI 1640 medium was used as a blank (*n* = 3). Cell-conditioned medium was collected and centrifuged at 2000× *g* for 25 min at 4 °C to pellet and remove cells, debris, and apoptotic bodies. The supernatant was filtered using a 0.22 μm pore PES filter (Merck Millipore, Burlington, MA, USA) to remove large sEVs. The filtrate was concentrated with a 100 kDa cut-off filter (Merck Millipore). The concentrate was ultracentrifuged at 37,000 rpm (average RCF is 234,700× *g*) for 70 min at 4 °C (SW41Ti rotor, Beckman Coulter, Brea, CA, USA). The pellet was washed with physiological saline (Hikari Pharmaceutical, Tokyo, Japan) and was collected by ultracentrifuge. This washing procedure was repeated twice. Finally, the weight of the pellet was adjusted to 0.05 g (≈ 50 μL) by adding physiological saline.

### 4.3. Extraction of Hydrophilic Metabolites from Cells

For metabolite extraction, 3.0 × 10^5^ PANC-1 cells were seeded in 60 mm dishes and cultured, as described above. To extract hydrophilic metabolites, cells were washed twice with 4 mL of ice-cold 5% (*w/v*) aqueous mannitol and 1 mL of methanol containing 20 μmol/L internal standards (methionine sulfone and camphor 10-sulfonic acid) was added to each dish. Four hundred microliter of sample solution was transferred into a glass jacket tube. The homogenate was mixed with 100 µL of chloroform. After centrifugation (2400× *g*, 10 min, 4 °C), 200 µL of the upper layer was transferred to a new glass vial. The supernatant was dried using a centrifugal concentrator. Dried samples were dissolved in 50 µL of 50% (*v/v*) aqueous acetonitrile and immediately used for hydrophilic metabolome analysis.

### 4.4. Extraction of Hydrophilic Metabolites from sEVs

Hydrophilic metabolites were extracted from 45 µL of sEV pellet suspension. The sEV pellet suspension was mixed with 200 µL of methanol containing 1 μmol/L internal standards (methionine sulfone and camphor 10-sulfonic acid) and 50 µL of chloroform. After centrifugation (2400× *g*, 10 min, 4 °C), 150 µL of the upper layer was transferred to a glass vial and concentrated by a centrifugal concentrator. Dried samples were dissolved in 20 µL of 50% (*v/v*) aqueous acetonitrile and immediately used for the analysis of the hydrophilic metabolome.

### 4.5. Extraction of Lipids from Cells

First, we prepared a lipid extraction solvent, supplemented with 10 µL of the mouse SPLASH^®^ LIPIDOMIX^®^ Mass Spec Internal Standard (Avanti Polar Lipids, Alabaster, AL, USA) containing (LPE 18:1 (d7), 2 μmol/L; PG 15:0–18:1 (d7) and PE (P) C18(Plasm)-18:1 (d9), 5 μmol/L; PE 15:0–18:1 (d7), 7 μmol/L; phosphatidic acid (PA) 15:0–18:1 (d7), 10 μmol/L; DG 15:0–18:1 (d7), 15 μmol/L; PS 15:0–18:1 (d7), PI 15:0–18:1 (d7), Alkenyl-acyl phosphatidylcholine (PC P) C18:0–18:1 (d9), and SM d18:1–18:1 (d9), 20 μmol/L; TG 15:0–18:1 (d7)-15:0, 35 μmol/L; LPC 18:1 (d7), 45 μmol/L; PC 15:0–18:1 (d7), 100 μmol/L; CE 18:1 (d7), 250 μmol/L), and 10 µL of internal standard mix (Cer d18:1–17:0, HexCer d18:1 (d5)-18:1, and FA 17:0, 10 μmol/L; MG 17:1, 100 μmol/L; cholesterol (d7), 300 μmol/L), and made up the volume to 1 mL with methanol.

The cells were washed twice with 4 mL of ice-cold D-PBS and then lysed in 700 µL of the lipid extraction solvent. The supernatant (400 µL) was mixed vigorously with 160 µL of methanol, 280 µL of chloroform, and 160 µL of Milli-Q water using a vortex mixer for 1 min followed by 5 min of sonication.

After centrifugation (16,000× *g*, 5 min, 4 °C), 800 µL of supernatant was transferred to a new tube. The supernatant was mixed vigorously with 220 µL of chloroform and Milli-Q water, and centrifuged (16,000× *g*, 5 min, 4 °C). The bottom layer (500 µL) was concentrated to dryness under a nitrogen stream. The dried samples were dissolved in 100 µL of 50% (*v/v*) chloroform/methanol for lipid analysis. Target lipids were determined using a pooled sample prepared with an equal amount of all cell and sEV samples.

### 4.6. Extraction of Lipids from sEVs

The lipid extraction solvent (560 µL) was added to 45 µL of sEV pellet suspension. The suspension was mixed vigorously with 280 µL of chloroform and 160 µL of Milli-Q water using a vortex mixer for 1 min followed by 5 min of sonication. The subsequent steps were the same as those described for cells.

### 4.7. Analysis of Hydrophilic Metabolites

Anionic metabolites were analyzed by capillary IC-MS, as previously described [19]. Briefly, capillary IC-MS was performed using a Dionex ICS-5000+ system coupled with a Q Exactive Orbitrap MS system (Thermo Fisher Scientific, San Jose, CA, USA). Separation of anionic metabolites was performed using a Dionex IonPac AS11-HC-4 µm column (250 × 0.4 mm, 4 µm; Thermo Fisher Scientific). The column temperature was maintained at 35 °C. The eluent flow rate was 20 µL/min and the KOH gradient was as follows: 1 mM, 0–2 min; 1 mM to 20 mM, 2–16 min; 20 mM to 100 mM, 16–35 min; 100 mM, 35–40 min; 100 mM to 1 mM, 40–40.1 min; 1 mM, 40.1–45.1 min. To enhance the ionization, isopropanol containing 0.1% acetic acid was delivered as the make-up solution at 5 µL/min. Sample injection volume was 0.4 µL. The Q Exactive mass spectrometer was operated in electrospray ionization (ESI) negative-ion mode. ESI parameters were as follows: sheath gas, 20 (arbitrary units); auxiliary gas, 10 (arbitrary units); sweep gas, 0; spray voltage, 4.0 kV; capillary temperature, 300 °C; S-lens, 35 (arbitrary units). Data were acquired in full MS scan mode and the parameters were as follows: resolution, 70,000; auto gain control target, 1 × 10^6^; maximum ion injection time, 100 ms; scan range, 70–1000 *m/z*.

Cationic metabolites were measured using LC-MS, as described previously [37]. Briefly, LC-MS analysis was performed using an Agilent 1290 Infinity LC system (Agilent Technologies, Santa Clara, CA, USA) equipped with a Q Exactive Orbitrap MS system. Separations were performed on a SeQuant^®^ ZIC^®^-pHILIC column (150 × 2.1 mm, 5 µm; EMD Millipore, Billerica, MA, USA). The mobile phase was composed of 10 mmol/L ammonium acetate, pH 9.8 (A) and acetonitrile (B). The flow rate was 0.25 mL/min, and the following linear gradient was used: 0–15 min, 90% to 30% B; 15–18 min, 30% B; 18–19 min, 30% to 90% B, followed by equilibration with 90% B for 15 min. The injection volume was 2 µL, and the column temperature was maintained at 20 °C. The Q Exactive mass spectrometer was operated in a heated ESI (HESI) positive-ion mode using the following source parameters: spray voltage = 3.0 kV, capillary temperature = 250 °C, sheath gas flow rate = 40 (arbitrary units), auxiliary gas flow rate = 10 (arbitrary units), auxiliary gas temperature = 300 °C, sweep gas flow rate = 0, S-lens = 35 (arbitrary units). Data were acquired in full MS scan mode and the parameters were as follows: resolution, 35,000; auto gain control target, 1 × 10^6^; maximum ion injection time, 100 ms; scan range, 70–1000 *m/z*.

### 4.8. Lipidomic Analyses

Lipids were measured by SFC-QqQMS, as previously described [20] but with modifications. Briefly, SFC-QqQMS experiments were performed using an Agilent 1260 Infinity II SFC system equipped with an Agilent 6470A triple quadrupole LC/MS system with an Agilent jet stream (AJS) ESI interface. Lipids were separated on an ACQUITY UPC2 Torus DEA column (3.0 × 100 mm, 1.7 µm; Waters, Milford, MA, USA) maintained at 60 °C. The mobile phase comprised supercritical carbon dioxide (A) and 0.1% (*w/v*) ammonium acetate in 95% (*v/v*) methanol (B). The flow rate of the mobile phase was 1.0 mL/min. The gradient of solution B was as follows: 1% from 0 min to 1 min, 75% at 24 min, 75% from 24 to 26 min, and 1% at 26.4 min; this was maintained until 30 min. The back pressure regulator was operated at 90 bar and 60 °C. The injection volume was 2 µL. To improve the ionization efficacy in ESI, mobile phase B was added as a make-up solution after the backpressure regulator at 0.05 mL/min.

AJS-ESI-MS/MS was operated in a positive/negative ion mode using the following source parameters: dry gas temperature = 300 °C, dry gas flow rate = 10 L/min, nebulizer pressure = 30 psi, sheath gas temperature = 350 °C, sheath gas flow rate = 12 L/min, capillary voltage = 3.0 kV, nozzle voltage = 0 V, fragmentor voltage = 380 V. Data were acquired using dynamic multiple reaction monitoring (MRM) mode, and the MRM transition setting was based on the in-house lipid MRM library described previously [20].

### 4.9. Data Analysis

The data acquired using capillary IC-MS and LC-MS were analyzed using the TraceFinder software (version 3.2, Thermo Fisher Scientific). The raw data obtained by SFC-QqQMS were analyzed using the MassHunter software (version 10.0, Agilent Technologies). To calculate the amounts of metabolites in sEVs and cells, the mol of individual metabolite species detected in the non-cultured medium sample and technical blank were subtracted from sEV and cell samples, respectively. The negative values were changed to zero. The amount of metabolites in sEVs was normalized to the number of particles, and that in cells was calculated with respect to the number of cells. Data of metabolites detected in 50% or more samples were used. Student’s *t*-test was used for statistical analysis. PCA was analyzed using SIMCA (version 13.0). The coefficient of determination and multiple correlation coefficients were analyzed using GraphPad Prism (version 8.4.3)

## Figures and Tables

**Table 1:** Top 20 hydrophilic metabolites in cells and small extracellular vesicles (sEVs) under normoxia.

	Cells (fmol/cell)		sEVs (pmol/10^11^ Particles)		
Rank	Metabolite	Amount	Metabolite	Amount	Cell Rank
1	**Phosphorylcholine**	628	**Phosphorylcholine**	18.0	1
2	Glutathione (reduced)	617	**Glycerophosphorylcholine**	10.2	5
3	**Glu**	447	Arg	5.7	24
4	**Ethanolamine phosphate**	294	**Glu**	4.7	3
5	**Glycerophosphorylcholine**	159	Lys	3.2	56
6	Asp	148	**Ethanolamine phosphate**	2.9	4
7	**Gln**	124	Inosine	2.8	−
8	**Gly**	122	**UDP-*N*-acetylglucosamine**	2.7	15
9	Pro	92.1	ADP	2.7	40
10	Lactic acid	77.0	**Gln**	2.6	7
11	ATP	49.9	Glucose 1-phosphate	2.6	95
12	Gly Gly	49.2	Ala	2.5	26
13	Asn	48.4	GDP	1.7	86
14	N-Acetylaspartate	32.2	UMP	1.7	35
15	**UDP-N-acetylglucosamine**	28.7	*N,N*-dimethylglycine	1.7	−
16	UTP	26.0	UDP-glucose	1.7	25
17	Citric acid	20.3	**Gly**	1.6	8
18	Creatine	20.0	Cytidine	1.6	−
19	beta-Ala	19.2	Uridine	1.6	−
20	Malic acid	18.4	UDP	1.6	63

The mean values of metabolites are shown. The names in bold font are of metabolites detected in both the samples. Cell rank indicates the rank among all the metabolites quantified in cells.

**Table 2:** Number of lipids of each class identified in cells and small extracellular vesicles (sEVs).

Lipid Class	Abbreviation	Number of Lipids Detected				
		Cells	sEVs	Common	Only Cells	Only sEVs
Free Fatty Acid	FA	3	3	1	2	2
Lysophosphatidylcholine	LPC	19	9	9	10	0
Lysophosphatidylethanolamine	LPE	14	15	14	0	1
Phosphatidylcholine	PC	80	50	48	32	2
Alkyl-Acyl Phosphatidylcholine/ Alkenyl-Acyl Phosphatidylcholine	PC (O)/ PC (P)	27	27	26	1	1
Phosphatidylethanolamine	PE	90	60	60	30	0
Alkenyl-Acyl Phosphatidylethanolamine	PE (P)	79	73	72	7	1
Phosphatidylglycerol	PG	16	9	9	7	0
Phosphatidic Acid	PA	17	15	15	2	0
Phosphatidylinositol	PI	82	53	53	29	0
Phosphatidylserine	PS	51	51	51	0	0
Sphingomyelin	SM	15	13	13	2	0
Ceramide	Cer	13	12	12	1	0
Hexosylceramides	HexCer	8	8	8	0	0
Cholesterol	Cholesterol	1	1	1	0	0
Cholesterol Ester	CE	5	6	5	0	1
Monoacylglycerol	MG	4	9	2	2	7
Diacylglycerol	DG	43	51	39	4	12
Triacylglycerol	TG	33	29	29	4	0
	Total	600	494	467	133	27

**Table 3:** Lipid content in cells and small extracellular vesicles (sEVs) under different oxygen conditions.

Lipid Class	Cells (amol/cell)							sEVs (pmol/10^11^ Particles)						
	Normoxia			Hypoxia			*p* Value	Normoxia			Hypoxia			*p* Value
	Mean	±	SD	Mean	±	SD		Mean	±	SD	Mean	±	SD	
Free Fatty Acid (FA)	18.2	±	6.7	9.4	±	12.9	0.3538	7.8	±	6.5	3.6	±	3.2	0.3631
Lysophosphatidylcholine (LPC)	83.2	±	10.4	151	±	20.9	0.0074	6.6	±	3.4	5.7	±	2.1	0.7302
Lysophosphatidylethanolamine (LPE)	104	±	17.9	193	±	25.9	0.0082	56.8	±	23.8	42.5	±	16.7	0.4420
Phosphatidylcholine (PC)	59338	±	8356	93637	±	11729	0.0146	3608	±	715	3388	±	860	0.7506
Alkyl-Acyl Phosphatidylcholine (PC (O))/ Alkenyl-Acyl Phosphatidylcholine (PC (P))	1689	±	213	2870	±	247	0.0033	452	±	105	379	±	91.5	0.4192
Phosphatidylethanolamine (PE)	13766	±	706	21431	±	279	0.0001	999	±	230	899	±	214	0.6105
Alkenyl-Acyl Phosphatidylethanolamine (PE (P))	11795	±	1200	22803	±	5034	0.0211	3792	±	910	2927	±	472	0.2172
Phosphatidylglycerol (PG)	243	±	20.7	397	±	39.5	0.0040	4.1	±	1.1	2.8	±	0.7	0.1570
Phosphatidic Acid (PA)	524	±	28.1	953	±	125	0.0043	161	±	30.8	153	±	37.1	0.8030
Phosphatidylinositol (PI)	5381	±	464	8851	±	468	0.0008	162	±	36.7	139	±	32.8	0.4580
Phosphatidylserine (PS)	5257	±	377	8850	±	491	0.0006	2038	±	352	1970	±	444	0.8456
Sphingomyelin (SM)	3279	±	220	5346	±	274	0.0005	1134	±	250	938	±	202	0.3501
Ceramide (Cer)	77.3	±	8.1	98.6	±	4.1	0.0152	7.2	±	2.1	5.2	±	1.2	0.2197
Hexosylceramides (Hexcer)	68.9	±	4.3	125	±	7.0	0.0003	32.3	±	5.9	31.6	±	6.5	0.8995
Cholesterol	23504	±	2817	36016	±	3118	0.0067	11080	±	2242	9346	±	2925	0.4608
Cholesterol Ester (CE)	704	±	172	739	±	152	0.8063	388	±	16.1	492	±	147	0.2923
Monoacylglycerol (MG)	10.5	±	4.5	21.6	±	8.6	0.1174	654	±	384	524	±	252	0.6509
Diacylglycerol (DG)	174	±	22.1	275	±	38.7	0.0172	49.9	±	14.5	42.1	±	10.1	0.4855
Triacylglycerol (TG)	1104	±	308	1755	±	457	0.1102	10.7	±	5.9	7.6	±	5.1	0.5210

The Student’s *t*-test was used to determine the *p* value.

## Data Availability

The data presented in this study are available on request from the corresponding author.

## Supplementary Materials

The following are available online at https://www.mdpi.com/article/10.3390/metabo11040215/s1, Supplementary Data S1: Materials and Methods. Figure S1. Validation of small extracellular vesicles (sEVs) derived from PANC-1 cells cultured under normoxia and hypoxia. Figure S2: Response of PANC-1 cells to hypoxia. Table S1. List of metabolites detected only in small extracellular vesicles (sEVs). Table S2. The KEGG pathways associated with top 20 hydrophilic metabolites in cells and small extracellular vesicles (sEVs) under normoxia. Table S3: Primer sequences for real-time PCR assays.
