# Supplementary material for: Metabolomic Analysis of Small Extracellular Vesicles Derived from Pancreatic Cancer Cells Cultured under Normoxia and Hypoxia
Source: Metabolites. 2021 Apr 1;11(4):215. doi: 10.3390/metabo11040215 (PMC8066639; doi:10.3390/metabo11040215)
Supplement: Supplementary file 1 [file metabolites-11-00215-s001.pdf]

Supporting information

Metabolomic Analysis of Small Extracellular Vesicles Derived from Pancreatic Cancer Cells Cultured under Normoxia and Hypoxia

This PDF file includes:
S1 Materials and Methods
Figure S1, S2
Table S1–3

S1. Materials and Methods

1.1. Isolation of Cellular RNA and mRNA Analyses

PANC-1 cells (ATCC, Manassas, VA, USA) were seeded in 6-well plates at 2.0 × 10^5 cells/well and precultured in RPMI 1640 (FUJIFILM Wako Pure Chemical Corporation, Osaka, Japan) containing 10% (*v*/*v*) fetal bovine serum (FBS, Biowest, Nuaillé, France), antibiotics (100 U/mL penicillin, 100 mg/mL streptomycin, and 0.25 mg/mL amphotericin B, Nacalai tesque, Kyoto, Japan) under normoxic conditions for 24 h. The cells were washed twice with Dulbecco's phosphate-buffered saline (D-PBS, Nacalai Tesque). Thereafter, the medium was changed to advanced RPMI 1640 medium (Thermo Fisher Scientific, Waltham, MA, USA) containing 2 mmol/L glutamine (Thermo Fisher Scientific) and antibiotics. The cells were then cultured for 48 h under hypoxic (1% O_2) or normal oxygen conditions. After washing with D-PBS, the cells were sampled and stored at −80 °C. mRNA was extracted with TRIzol Reagent (Thermo Fisher Scientific) according to the manufacturer's protocol. For cDNA synthesis, a cDNA synthesis kit (ReverTra Ace α, Toyobo, Osaka, Japan) was used. Quantitative real-time PCR was performed using TB Green *Premix Ex Taq II* (Takara Bio, Shiga, Japan) on a StepOnePlus Real-time PCR system (Thermo Fisher Scientific) according to the manufacturer's instructions. Quantitation was performed using the ΔΔCt method, with the expression of *RPL27* used as an internal reference. The primers used for real-time PCR are shown in Table S3.

1.2. Immunoblot Analysis

Whole cells and sEVs were extracted using M-PER reagent (Thermo Fisher Scientific) containing protease inhibitor cocktails (Roche, Basel, Switzerland). Protein concentration in cells was measured by the Bradford method (Quick Start™ Bradford 1x Dye Reagent, Bio-Rad, Hercules, CA, USA). Protein concentration in sEVs was measured by Micro BCA™ Protein Assay Kit (Thermo Fisher Scientific). Equal amounts of each of protein samples were separated on a 4%–15% Mini-PROTEAN® TGX™ precast protein gel (Bio-Rad) and transferred to a PVDF membrane (Bio-Rad) using the Trans-Blot® Turbo™ Transfer System (Bio-Rad). Membranes were blocked with the Blocking One reagent (Nacalai Tesque). Antibodies specific for glucose transporter 1 (GLUT1) (ab14683, Abcam, Cambridge, UK), anti-β-actin (sc-47778, Santa Cruz, Dallas, TX, USA), CD63 (ab8219, Abcam), CD81 (ANC-302-020, Ancell, Stillwater, MN, USA), Syntenin-1 (ab133267, Abcam), and Calnexin (Cell signaling Technology, Danvers, MA, USA) were used as primary antibodies. HRP-labeled anti-rabbit IgG antibody (Cytiva Marlborough, MA, USA) and anti-mouse IgG antibodies (Cytiva) were used as the secondary antibodies. The membranes were subjected to chemiluminescent analysis using the Clarity Western ECL Substrate (Bio-Rad) and the images were analyzed using the Image Quant LAS 4000 mini software (Cytiva).

1.3. Nanoparticle Tracking Analysis

We measured the number of particles and size distribution by nanoparticle tracking analysis (NanoSight LM10, Malvern Analytical, Malvern, UK) (biological $n = 3$, technical $n = 3$).

1.4. miRNA Analysis of sEVs

Isolation of exosomal miRNAs was performed using the miRNeasy Mini Kit (Qiagen, Venlo, Netherlands). The sEV pellets were dissolved in 100 μL of physiological saline. Five hundred microliters of QIAzol lysis reagent (Qiagen) was added to the sample. After 5-min of incubation, 5 μL of 100 pmol/L s*yn-cel-miR-39* (Qiagen) was added to the tube as a spike-in control for losses in preparation. The subsequent steps were performed according to the manufacturer's instructions. For cDNA synthesis, the Taqman MicroRNA Reverse Transcription Kit (Thermo Fisher Scientific) was used. Quantitative real-time PCR was performed using the TaqMan MicroRNA Assay (Thermo Fisher Scientific) and TaqMan Universal PCR Master Mix, no AmpErase UNG (Thermo Fisher Scientific) on a StepOnePlus Real-time PCR system (Applied Biosystems) according to the manufacturer's instructions. Quantitation was performed using the ΔΔCt method, with synthetic spike control (*syn-cel-miR-39*) used as an invariant control. miRNA data were normalized by the number of particles determined by NanoSight analysis.

Transmission Electron Microscopy

Transmission electron microscopy (TEM) analysis was performed by Tokai Electron Microscopy, Inc. The sEV samples were absorbed onto carbon-coated copper grids (400 mesh) and were stained with 2% phosphotungstic acid solution (pH 7.0) for 15 s. The grids were observed using a transmission electron microscope (JEM-1400 plus, JEOL Ltd., Tokyo, Japan) at an acceleration voltage of 100 kV. Digital images were taken with a CCD camera (EM-14830RUBY2, JEOL Ltd.).

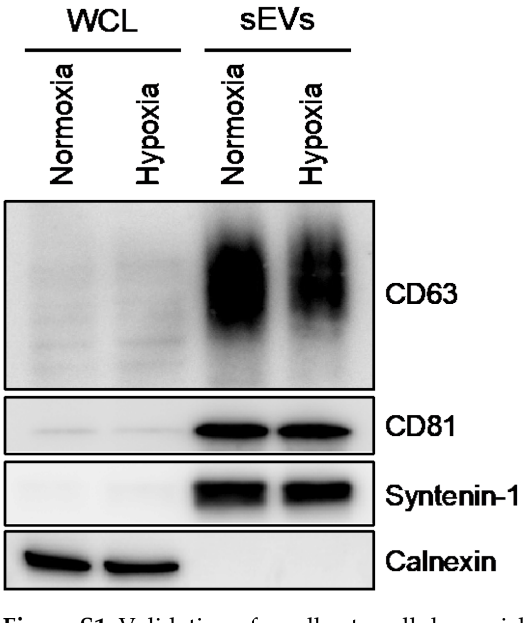

Figure S1. Validation of small extracellular vesicles (sEVs) derived from PANC-1 cells cultured under normoxia and hypoxia. Immunoblotting of CD63, CD81, syntenin-1, and calnexin in PANC-1 cells and sEVs under normoxia and hypoxia. sEV markers, CD63, CD81, and syntenin-1; EV negative protein marker, calnexin. The protein content was 10 μg for the whole cell lysate (WCL) sample and 1.5 μg for the sEV sample.

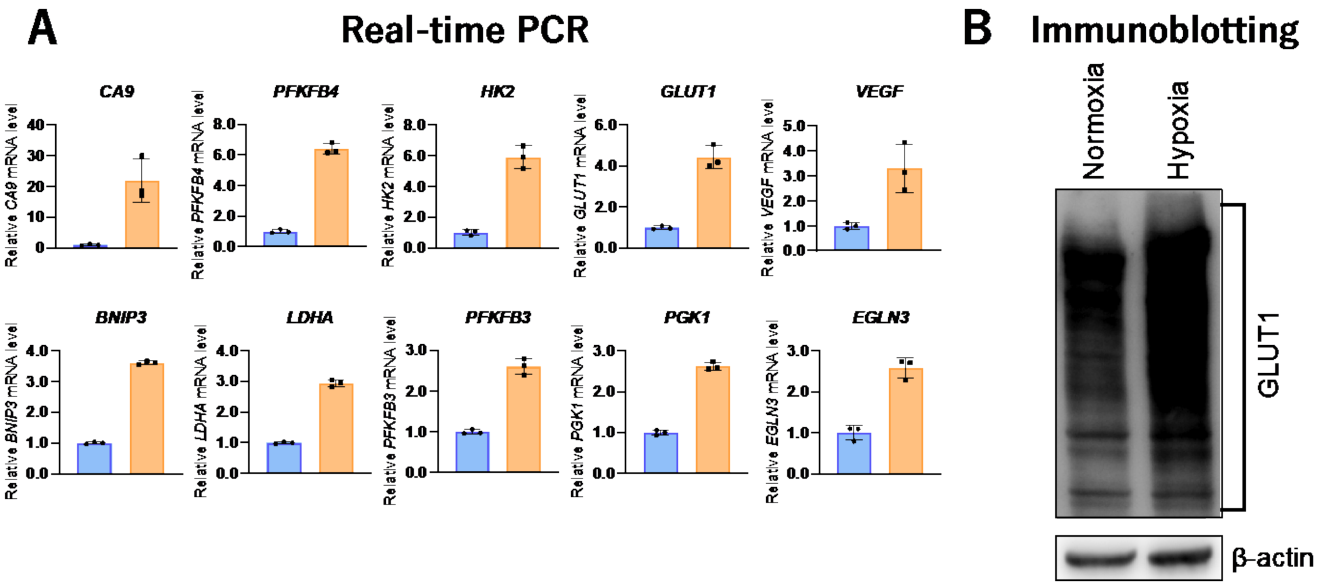

Figure S2. Response of PANC-1 cells to hypoxia. (**A**) Expression of hypoxia-related mRNAs in PANC-1 cells under normoxia (blue) and hypoxia (orange). Gene expression was determined by real-time PCR. Values are normalized to normoxia. (**B**) Immunoblotting of GLUT1 in PANC-1 cells under normoxia and hypoxia. β-Actin was used as a loading control.

Table S1. List of metabolites detected only in small extracellular vesicles (sEVs).

Metabolite	KEGG Pathway
Inosine	Purine metabolism
N,*N*-dimethylglycine	Glycine, serine, and threonine metabolism
Cytidine	Pyrimidine metabolism
Uridine	Pyrimidine metabolism
Guanosine	Purine metabolism
Citrulline	Arginine biosynthesis
Hypoxanthine	Purine metabolism
Gly Leu	—
Xanthine	Purine metabolism
Hexylamine	—
2-Deoxyribose 1-phosphate	Pyrimidine metabolism, Pentose phosphate pathway

Table S2. The KEGG pathways associated with top 20 hydrophilic metabolites in cells and small extracellular vesicles (sEVs) under normoxia.

	Cells		sEVs	
**Rank**	**Metabolite**	**KEGG pathway**	**Metabolite**	**KEGG Pathway**
1	Phosphorylcholine	Glycerophospholipid metabolism, Choline metabolism in cancer	Phosphorylcholine	Glycerophospholipid metabolism, Choline metabolism in cancer
2	Glutathione (reduced)	Glutathione metabolism, Cysteine and methionine metabolism	Glycerophosphorylcholine	Glycerophospholipid metabolism, Choline metabolism in cancer
3	Glu	Alanine, aspartate, and glutamate metabolism	Arg	Arginine and proline metabolism
4	Ethanolamine phosphate	Glycerophospholipid metabolism	Glu	Alanine, aspartate, and glutamate metabolism
5	Glycerophosphorylcholine	Glycerophospholipid metabolism, Choline metabolism in cancer	Lys	Lysine biosynthesis
6	Asp	Alanine, aspartate, and glutamate metabolism	Ethanolamine phosphate	Glycerophospholipid metabolism
7	Gln	Alanine, aspartate, and glutamate metabolism, Central carbon metabolism in cancer	Inosine	Purine metabolism
8	Gly	Glycine, serine, and threonine metabolism	UDP-*N*-acetylglucosamine	Amino sugar and nucleotide sugar metabolism
9	Pro	Arginine and proline metabolism	ADP	Oxidative phosphorylation, Purine metabolism
10	Lactic acid	Glycolysis, Gluconeogenesis, Central carbon metabolism in cancer	Gln	Alanine, aspartate, and glutamate metabolism, Central carbon metabolism in cancer
11	ATP	Oxidative phosphorylation, Purine metabolism	Glucose 1-phosphate	Glycolysis, Pentose, and glucuronate interconversions
12	Gly Gly	—	Ala	Alanine, aspartate, and glutamate metabolism, Cysteine and methionine metabolism
13	Asn	Alanine, aspartate, and glutamate metabolism	GDP	Purine metabolism
14	N-Acetylaspartate	Alanine, aspartate, and glutamate metabolism	UMP	Pyrimidine metabolism
15	UDP-N-acetylglucosamine	Amino sugar and nucleotide sugar metabolism	*N*,*N*-dimethylglycine	Glycine, serine, and threonine metabolism
16	UTP	Pyrimidine metabolism	UDP-glucose	Pentose and glucuronate interconversions
17	Citric acid	Citrate cycle (TCA cycle), Central carbon metabolism in cancer	Gly	Glycine, serine, and threonine metabolism
18	Creatine	Glycine, serine, and threonine metabolism, Arginine and proline metabolism	Cytidine	Pyrimidine metabolism
19	beta-Ala	beta-Alanine metabolism, Propanoate metabolism	Uridine	Pyrimidine metabolism
20	Malic acid	Citrate cycle (TCA cycle), Central carbon metabolism in cancer	UDP	Pyrimidine metabolism

Table S3. Primer sequences for real-time PCR assays.

Gene Name	**Forward Primer Sequence (5′-3′)**	**Reverse Primer Sequence (5′-3′)**
GLUT1	ATCGTCGTCGGCATCCTCAT	TGTCCCGCGCAGCTTCTTTA
LDHA	TATCTTGACCTACGTGGCTT	CATTAGGTAACGGAATCGGG
VEGF	ACCATGAACTTTCTGCTGTC	TACTCCTGGAAGATGTCCAC
CA9	GGATCTACCTACTGTTGAGGCT	CATAGCGCCAATGACTCTGGT
EGLN3	CTGGGCAAATACTACGTCAAGG	GACCATCACCGTTGGGGTT
HK2	GAGCCACCACTCACCCTACT	CCAGGCATTCGGCAATGTG
PGK1	TGGACGTTAAAGGGAAGCGG	GCTCATAAGGACTACCGACTTGG
PFKFB3	TTGGCGTCCCCACAAAAGT	AGTTGTAGGAGCTGTACTGCTT
PFKFB4	TCCCCACGGGAATTGACAC	GGGCACACCAATCCAGTTCA
BNIP3	CAGGGCTCCTGGGTAGAACT	CTACTCCGTCCAGACTCATGC
RPL27	CTGTCGTCAATAAGGATGTCT	CTTGTTCTTGCCTGTCTTGT
